# Inspiratory flow profile and usability of the NEXThaler, a multidose dry powder inhaler, in asthma and COPD

**DOI:** 10.1186/s12890-021-01430-9

**Published:** 2021-02-25

**Authors:** Alfredo Chetta, Arzu Yorgancioglu, Mario Scuri, Sara Barile, Daniele Guastalla, P. N. Richard Dekhuijzen

**Affiliations:** 1 Respiratory Disease and Lung Function Unit, Department of Medicine and Surgery, University of Parma, Parma, Italy; 2 Department of Pulmonology, Medical Faculty, Celal Bayar University, Manisa, Turkey; 3 Chiesi Farmaceutici SpA, Parma, Italy; 4 Radboud University Medical Center, Nijmegen, The Netherlands

**Keywords:** Dry powder inhalers, Asthma, Chronic obstructive pulmonary disease, Inspiratory flow, Breath-actuated mechanism

## Abstract

**Background:**

Inhaler selection is important when managing respiratory conditions; a patient’s inhalation technique should be appropriate for the selected device, and patients should ideally be able to use a device successfully regardless of disease severity. The NEXThaler is a multidose dry-powder inhaler with a breath-actuated mechanism (BAM) and dose counter that activates only following inhalation, so effectively an ‘inhalation counter’. We assessed inspiratory flow through the NEXThaler in two studies and examined whether inhalation triggered the BAM.

**Methods:**

The two studies were open-label, single-arm, and single visit. One study recruited patients with asthma aged ≥ 18 years; the other recruited patients with chronic obstructive pulmonary disease (COPD) aged ≥ 40 years. All patients inhaled twice through a placebo NEXThaler. The inspiratory profile through the device was assessed for each inhalation using acoustic monitoring, with flow at and time to BAM firing, peak inspiratory flow (PIF), and total inhalation time assessed.

**Results:**

A total of 40 patients were enrolled in the asthma study: 20 with controlled asthma and 20 with partly controlled/uncontrolled asthma. All patients were able to trigger the BAM, as evidenced by the inhalation counter activating on closing the device. Mean flow at BAM firing following first inhalation was 35.0 (range 16.3–52.3) L/min; mean PIF was 64.6 (35.0–123.9) L/min. A total of 72 patients were enrolled in the COPD study, with data analysed for 69 (mean forced expiratory volume in 1 s 48.7% predicted [17–92%]). As with the asthma study, all patients, regardless of airflow limitation, were able to trigger the BAM. Mean flow at BAM firing following first inhalation was 41.9 (26.6–57.1) L/min; mean PIF was 68.0 (31.5–125.4) L/min. Device usability was rated highly in both studies, with 5 min sufficient to train the patients, and a click heard shortly after inhalation in all cases (providing feedback on BAM firing).

**Conclusions:**

Inhalation flows triggering the BAM in the NEXThaler were similar between patients with controlled and partly controlled/uncontrolled asthma, and were similar across COPD airflow limitation. All enrolled patients were able to activate the device.

**Supplementary Information:**

The online version contains supplementary material available at 10.1186/s12890-021-01430-9.

## Background

Inhaler selection is an important consideration in overall therapy choice when managing respiratory conditions [1, 2], and the choice of device should be tailored to the patient [3]. The ideal inhaler should be breath-activated, should provide feedback that the inhalation manoeuvre has been successful [4], and should indicate the number of remaining doses [5]. In addition, a patient’s inhalation technique should be appropriate for the device [6]. Successful use of a dry-powder inhaler (DPI) requires that a patient can generate sufficient inspiratory flow [6]; ideally patients with all levels of disease severity should be able to use a device successfully.

The NEXThaler is a multidose DPI with a breath-actuated mechanism (BAM) that has an inspiratory flow resistance of 0.036 kPa^½^ L/min (i.e., medium-to-high resistance). To inhale a dose of medication, the patient only has to open the cover, which makes the dose available. When the patient then inhales through the device, a click is felt or heard as the BAM fires, indicating successful dose release. Then, when the cover is closed, if the dose has been inhaled the counter will count down. If the inhalation was not successful, for example if the patient did not generate sufficient inspiratory flow, just exhaled through the device, or only opened and closed the cover, the counter will not count down, and the dose is not wasted. Data on dose- and flow-independency of the NEXThaler across various inspiratory flows have previously been published [7, 8], as have lung deposition [9] and usability data [10].

The NEXThaler was designed to have an inspiratory flow at BAM firing of 35 L/min. In this manuscript, we describe the results of two studies that assessed the actual inspiratory flow profile generated by patients with asthma and chronic obstructive pulmonary disease (COPD) through the NEXThaler device, and examined whether this manoeuvre triggered the BAM. The studies also evaluated device usability as perceived by the patients.

## Methods

### Trial design

The two studies were of similar design, being open-label, single-arm, and single visit, with both conducted at a single centre (a specialist investigation unit in Italy). Males or females were eligible. Other than the diagnosis, the main differences in inclusion criteria were age (minimum 18 years for the asthma study; 40 years for the COPD study) and that the COPD study recruited current or ex-smokers (there were no smoking-related criteria in the asthma study). Both studies excluded patients with a diagnosis of any restrictive lung disease. Other main reasons for exclusion from the asthma study were significant seasonal variation in their symptoms, asthma that occurred only during episodic exposure to an allergen, or a history of near-fatal asthma; the main exclusion criterion for the COPD study was a diagnosis of asthma. All patients provided written informed consent prior to any study-related procedure. The studies were approved by an independent ethics committee (Ospedale Maggiore di Parma), and were performed in accordance with the principles of the Declaration of Helsinki, and the International Conference on Harmonisation notes for guidance on Good Clinical Practice (ICH/CPMP/135/95). Both studies were registered in the EudraCT database (asthma study: registration number 2012-000039-22, registered 18 Jun 2012; COPD study: registration number 2013-000262-11, registered 16 May 2013).

At the single study visit, patients were initially trained on correct use using an empty NEXThaler DPI. The instructions for use during this training were as follows. “Hold your inhaler firmly in the upright position. Open the cover fully, check the dose counter window. Before inhaling, breathe out as far as comfortable; do not breathe out through the inhaler. Bring the inhaler up to your mouth and place your lips around the mouthpiece. Do not cover the air vent when holding the inhaler. Do not inhale through the air vent. Take a deep and forceful breath through your mouth. On inhalation check that an audible click is heard. Remove the inhaler from your mouth. Hold your breath for 5–10 s or as long as is comfortable. Breathe out. Move the inhaler back to the upright position and close the cover fully. Check that the counter has gone down by one.” All patients then inhaled twice through a placebo NEXThaler device, with the inhalations separated by a maximum of 5 min. The inspiratory profile through the device was assessed for each inhalation using acoustic monitoring (Sensohaler, Sagentia Inc., Cambridge, UK), with acoustic signals recorded using a condenser microphone placed within the device located so as not to impact the operation of the device, and then analysed by specialised software using a set of algorithms. An additional manoeuvre could be performed if the first manoeuvre was judged unacceptable by the investigator (for example if the microphone disconnected during the inhalation, there was an operator-related error in recording, or artifacts). The acoustic monitoring was used to assess the flow at and time to BAM firing, peak inspiratory flow (PIF), total inhalation time, and total inhaled volume, all of which were automatically calculated by the software. The overall performance of the acoustic monitoring system was validated by comparing the flow measured by the system against a completely independent flow measurement by a calibrated hot-wire anemometer. The estimated flow accuracy was within ± 5 L/min of the actual flow from 30 to 130 L/min and within ± 10 L/min from 0 to 30 L/min. Device usability was assessed by means of a physician-assessed questionnaire that comprised 10 questions (see supplement), each with a yes/no answer, with the responses entered directly in the patient’s case report form.

The primary objective of both studies was to assess the inspiratory profile through the NEXThaler, in patients with different levels of asthma control, or patients with COPD who had varying levels of airflow limitation.

### Sample size and statistical methods

There was no formal sample size calculation for either study. For the asthma study, 40 patients were considered to be sufficient to assess inhalation profile through the device, 20 with controlled asthma, and 20 with partly controlled asthma (where ‘controlled’ was defined as occurrence of daytime symptoms and rescue medication use twice or less per week, no limitation of activities, no nocturnal symptoms or awakening, and normal lung function [pre-bronchodilator forced expiratory volume in 1 s [FEV_1_] or peak expiratory flow [PEF] ≥ 80% predicted]). For the COPD study, the aim was to recruit 10–20 patients with Global Initiative for Chronic Obstructive Lung Disease (GOLD) Grade 1 airflow limitation (post-bronchodilator FEV_1_ ≥ 80% predicted), and 20 patients with Grades 2 to 4 (Grade 2, 50–80%; Grade 3 30–50%; Grade 4 < 30%). Assuming a screening failure rate of 10%, it was estimated that 44 patients would need to be screened for the asthma study, and 89 for the COPD study. Descriptive statistics are provided for each variable, analysed separately for the first and second inhalation.

## Results

### Asthma

#### Participants

A total of 40 patients were enrolled: 20 with controlled asthma and 20 with partly controlled or uncontrolled asthma. The mean ± SD age of these enrolled patients was 43.2 ± 16.5 years (range 18–77 years); 60% were female. Mean FEV_1_ at screening was 92.5 ± 12.5% predicted (95.1 ± 9.8% in the controlled asthma group and 89.8 ± 14.5% in the partly controlled/uncontrolled group), with mean PEF 91.6 ± 13.1% predicted (95.1 ± 11.3 and 88.0 ± 14.2%, respectively), ranging from 60.9 to 116.0%.

#### Outcomes

**Fig. 1:**
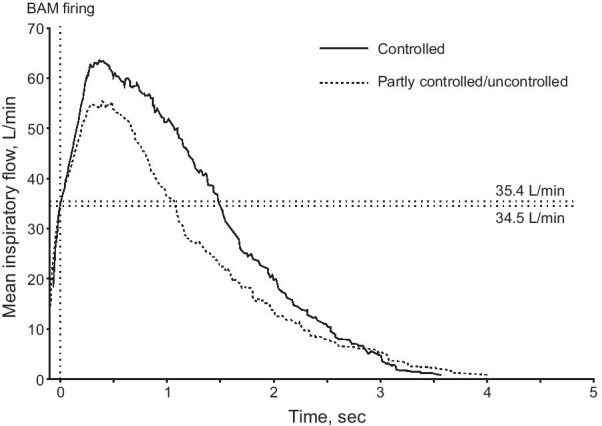
Asthma study: Mean inspiratory flow over time by asthma control (first inhalation). Values on the right of the plot area are the mean flow at BAM firing in patients with controlled (top) and partly controlled/uncontrolled asthma (bottom). *BAM* breath-actuated mechanism

**Fig. 2:**
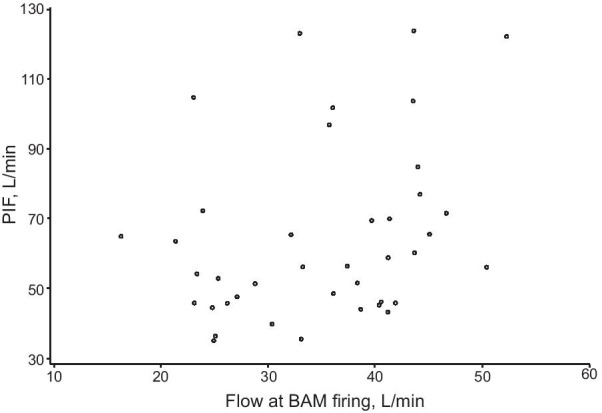
Asthma study: scatter plot of PIF versus flow at BAM firing (first inhalation). *PIF* peak inspiratory flow, *BAM* breath-actuated mechanism

All patients were able to trigger the BAM, as evidenced by the inhalation counter activating on closing the device. Inspiratory flow through the NEXThaler was higher in patients with controlled asthma than in those with partly controlled or uncontrolled asthma, both by individual timepoint (Fig. 1) and by PIF (Table 1). However, mean flows at BAM firing were similar, both for the two groups and the two inhalations (Table 1), and all patients were able to generate a PIF above the value required to fire the BAM (data from the first inhalation are shown in Fig. 2). Furthermore, the mean time to BAM firing was similar in the two groups and for the two inhalations (Table 1). Usability was rated highly, with 5 min sufficient to train all patients on device use, a click heard on opening and soon after inhalation, and the mouthpiece fitting well; the inhalation counter appearance was considered clear by all but one patient (Additional file 1: Table S1). Given the air vent is just next to the mouthpiece on the NEXThaler, we included a question specifically on whether the patients blocked these vents during use; none blocked the vents.

**Table 1:** Asthma study: inspiratory flow profile from acoustic monitoring

	Asthma control		Total (N = 40)
	Controlled (N = 20)	Partly controlled/ uncontrolled (N = 20)	
Flow at BAM firing, L/min			
First inhalation	35.4 ± 9.5 (16.3–52.3)	34.5 ± 8.6 (23.1–50.4)	35.0 ± 9.0 (16.3–52.3)
Second inhalation	34.6 ± 9.2 (14.4–50.8)	36.2 ± 9.0 (18.0–53.6)	35.4 ± 9.0 (14.4–53.6)
PIF, L/min			
First inhalation	70.5 ± 28.2 (39.9–123.9)	58.8 ± 20.1 (35.0–123.3)	64.6 ± 24.9 (35.0–123.9)
Second inhalation	72.1 ± 25.6 (42.1–117.6)	63.0 ± 16.8 (44.2–117.4)	67.6 ± 21.9 (42.1–117.6)
Time to BAM firing, s			
First inhalation	0.06 (0.02–0.50)	0.08 (0.03–0.50)	0.07 (0.02–0.50)
Second inhalation	0.07 (0.02–0.41)	0.08 (0.03–0.18)	0.07 (0.02–0.41)
Time to PIF, s			
First inhalation	0.47 (0.32–1.18)	0.50 (0.35–2.34)	0.48 (0.32–2.34)
Second inhalation	0.46 (0.32–1.40)	0.56 (0.36–1.22)	0.48 (0.32–1.40)
Total inhalation time, s			
First inhalation	1.93 (0.39–3.62)	1.80 (0.78–4.09)	1.83 (0.39–4.09)
Second inhalation	2.00 (0.49–3.49)	1.68 (0.67–3.54)	1.82 (0.49–3.54)
Total inhaled volume, L			
First inhalation	1.73 ± 0.96 (0.23–4.09)	1.39 ± 0.55 (0.50–2.67)	1.56 ± 0.79 (0.23–4.09)
Second inhalation	1.68 ± 0.84 (0.28–3.89)	1.39 ± 0.55 (0.45–2.63)	1.53 ± 0.72 (0.28–3.89)

Data are mean ± SD (range) for flow at BAM firing, PIF, and total inhaled volume, and median (range) for time to BAM firing, time to PIF, and total inhalation time

*BAM* breath-actuated mechanism, *PIF* peak inspiratory flow

### COPD

#### Participants

A total of 72 patients were enrolled: 21 with post-bronchodilator FEV_1_ ≥ 80% predicted (GOLD Grade 1); 20 with FEV_1_ 50–80% (Grade 2); 21 with FEV_1_ 30–50% predicted (Grade 3); and 10 with FEV_1_ < 30% predicted (Grade 4). Three patients were excluded from the analyses (two did not have inhalation profiles available [one GOLD Grade 1 and one Grade 3], and one due to an inaccurate COPD diagnosis [Grade 1]). For the remaining 69 patients, mean ± SD age was 67.9 ± 8.3 years (range 44–80 years), 58 (84.1%) were male, 21 (30.4%) were current smokers, post-bronchodilator FEV_1_ was 48.7 ± 20.0% predicted (range 17–92%), with post-bronchodilator FEV_1_ to forced vital capacity ratio 0.52 ± 0.11 (0.22–0.69).

#### Outcomes

**Fig. 3:**
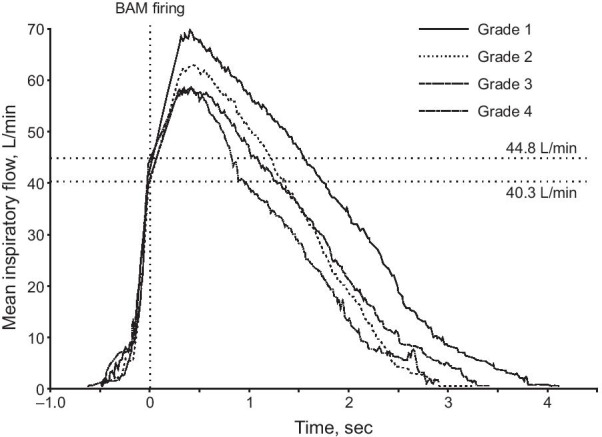
COPD study: mean inspiratory flow over time by GOLD Grade (first inhalation). Values on the right of the plot area are the minimum and the maximum mean flows at BAM firing across GOLD Grades. *GOLD* Global Initiative for Chronic Obstructive Lung Disease, *BAM* breath-actuated mechanism

**Fig. 4:**
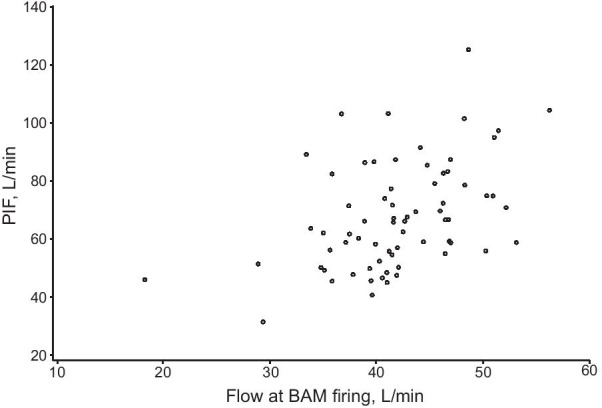
COPD study: scatter plot of PIF versus flow at BAM firing (first inhalation). *PIF* peak inspiratory flow, *BAM* breath-actuated mechanism

All patients, regardless of airflow limitation, were able to trigger the BAM, as evidenced by the inhalation counter being activated on closing the device. Inspiratory flow through the device was consistent with the degree of airflow limitation, being slightly higher in patients with GOLD Grades 1 and 2 than Grades 3 and 4 (Fig. 3 and Table 2). Importantly, however, all patients had a PIF above the BAM firing value on both inhalations, indicating that they were able to use the device effectively, regardless of airflow limitation (data from the first inhalation are shown in Fig. 4).

**Table 2:** COPD study: inspiratory flow profile from acoustic monitoring

	GOLD Grade				Total (N = 69)
	1 (N = 19)	2 (N = 20)	3 (N = 20)	4 (N = 10)	
Flow at BAM firing, L/min					
First inhalation	42.9 ± 6.4 (28.8–52.1)	40.3 ± 4.2 (33.8–50.9)	41.1 ± 7.0 (18.1–51.4)	44.8 ± 7.5 (29.2–56.2)	41.9 ± 6.3 (18.1–56.2)
Second inhalation	43.6 ± 7.4 (32.5–57.1)	40.8 ± 5.6 (26.6–47.4)	43.4 ± 5.2 (34.1–56.5)	41.0 ± 6.9 (30.1–48.6)	42.4 ± 6.2 (26.6–57.1)
PIF, L/min					
First inhalation	74.1 ± 20.8 (47.7–125.4)	69.1 ± 17.7 (45.3–103.4)	63.5 ± 15.0 (40.5–97.5)	63.5 ± 20.2 (31.5–104.5)	68.0 ± 18.4 (31.5–125.4)
Second inhalation	71.9 ± 22.1 (45.1–135.9)	70.5 ± 12.9 (45.4–90.7)	66.4 ± 17.8 (41.3–101.7)	55.3 ± 14.3 (32.0–82.3)	67.6 ± 17.9 (32.0–135.9)
Time to BAM firing, s					
First inhalation	0.10 (0.06–0.49)	0.14 (0.05–0.49)	0.16 (0.02–0.63)	0.14 (0.07–0.49)	0.13 (0.02–0.63)
Second inhalation	0.21 (0.06–0.53)	0.13 (0.05–0.31)	0.16 (0.07–0.72)	0.15 (0.05–0.31)	0.16 (0.05–0.72)
Time to PIF, s					
First inhalation	0.58 (0.38–1.56)	0.54 (0.36–1.17)	0.54 (0.11–1.46)	0.53 (0.42–0.99)	0.54 (0.11–1.56)
Second inhalation	0.56 (0.38–1.70)	0.60 (0.36–1.31)	0.63 (0.11–1.37)	0.45 (0.19–1.05)	0.59 (0.11–1.70)
Total inhalation time, s					
First inhalation	2.73 (1.86–4.24)	2.38 (0.96–3.34)	2.33 (0.98–4.03)	2.21 (0.77–3.20)	2.51 (0.77–4.24)
Second inhalation	2.68 (1.87–4.30)	2.33 (1.18–3.50)	2.40 (1.45–3.78)	2.11 (0.46–3.09)	2.44 (0.46–4.30)
Total inhaled volume, L					
First inhalation	2.28 ± 0.67 (1.27–4.05)	1.70 ± 0.60 (0.51–2.66)	1.75 ± 0.73 (0.38–3.65)	1.51 ± 0.62 (0.31–2.26)	1.85 ± 0.70 (0.31–4.05)
Second inhalation	2.15 ± 0.76 (1.18–4.36)	1.73 ± 0.54 (0.68–2.42)	1.73 ± 0.56 (0.65–2.83)	1.23 ± 0.62 (0.17–1.97)	1.78 ± 0.67 (0.17–4.36)

Data are mean ± SD (range) for flow at BAM firing, PIF, and total inhaled volume, and median (range) for time to BAM firing, time to PIF, and total inhalation time

*GOLD* Global Initiative for Chronic Obstructive Lung Disease, *BAM* breath-actuated mechanism, *PIF* peak inspiratory flow

The slope of the curves at the start of inhalation (indicating flow acceleration) was steep and consistent across GOLD Grades (Fig. 3). Furthermore, both the flow at and time to BAM firing were consistent across all GOLD Grades, and similar for the first and second inhalations, indicating highly consistent and reproducible device performance, independent of the severity of airflow limitation (Table 2). In the usability questionnaire, in all cases the inhalation counter appearance was clear, a click was heard on opening the inhaler and after drug inhalation and 5 min was sufficient time to train patients in the use of the device (Additional file 1: Table S2).

## Discussion

All patients in both studies were able to generate sufficient inspiratory flow to trigger the BAM, with firing occurring with a flow as low as 14.4 L/min in the asthma study and 18.1 L/min in the COPD study. The mean flow at BAM was similar in the two asthma groups, and was similar across the four airflow limitation categories in the COPD study, as was the time to BAM firing, indicating that disease characteristics did not impact the ability to trigger dose delivery in either disease. Furthermore, the mean time to BAM firing was early in the inhalation manoeuvre. Importantly, the inhalation counter activated on closure for all patients, confirming that all patients were able to activate the device, and a click was heard soon after inhalation in all cases, providing additional confirmation of BAM firing.

Interestingly, the inspiratory flows generated through the NEXThaler in the COPD study were overall larger than those in the asthma study. Although this may appear counter-intuitive (and differs from data in two previous studies with different DPIs [11, 12]), there was no requirement for bronchodilator washout prior to the use of the NEXThaler, so comparing the two populations is difficult, as is comparing these data with standard populations. Furthermore, our data are from two different studies (even though the designs of the studies were very similar), and no formal comparisons between the studies have been performed. In addition, the data are the peak flows achieved through the device, rather than peak flows generated through a spirometer, and so the data could be influenced by the instructions for use and the characteristics of the device itself.

The combination of the BAM and the dose counter only being activated following adequate inhalation means that the dose counter on the NEXThaler is effectively an ‘inhalation counter’ providing feedback on successful dose release. This is a key distinction between the NEXThaler and the Ellipta and the Turbuhaler multi-dose DPIs [13]. In these other devices, the inhalation counter is activated on priming, so that if the patient does not use the dose (either deliberately or accidentally) it is wasted [14, 15]—but more importantly this does not give any indication of correct use. In addition, although we did not evaluate dose delivery in the current studies (given we used placebo devices), a previous in-vitro study has demonstrated flow independency through the NEXThaler for inspiratory flows ranging from 30 to 90 L/min (at inhalation volumes of 2 and 4 L), with delivered doses remaining with a variation largely within ± 15% of the specification value (although the fine particle fraction, expressed as a percentage of the delivered dose, and the fine particle mass both increased with increasing flow) [8]. This is an important consideration given the impact of disease characteristics on the mean inspiratory flow that we observed in these two studies, and given we observed a range in PIF of 35.0–117.6 L/min in the asthma study and 31.5–135.9 L/min in the COPD study. A similar impact of disease characteristics on inspiratory flow through DPIs has been observed at least one other study [16], whereas two studies in patients with COPD showed no clear correlation using the Diskus, Ellipta, HandiHaler and Easyhaler DPIs [17, 18]. These contrasting results emphasise the importance of flow independency of dose delivery, as it is potentially difficult to predict the flow that a patient can generate through a device purely based on their disease characteristics.

The usability assessments in these two studies were consistent with the results of a previous study conducted in patients with asthma, in which the usability of the NEXThaler was compared with the Diskus and Turbuhaler [10]. In this previous study, in addition to overall ease of use the NEXThaler was rated by patients as superior to the two other DPIs in terms of the time to set up, and to read the instructions for use, and the proportion of participants who completed an error-free successful inhalation was significantly higher for the NEXThaler. We acknowledge that the data from these two studies are from evaluations performed immediately after patients were trained on correct device use. These data therefore don’t necessarily reflect ‘real world’ use. However, given the dose counter only activates following successful inhalation, if a patient did not generate sufficient inspiratory flow during a manoeuvre this will be immediately apparent. The wide variability of flow at BAM firing could be because activation occurred early in the inhalation manoeuvre (after a mean of 0.07 s in the asthma study and 0.13–0.16 s in the COPD study), and which is at the steepest part of the flow–time curve when the flow is increasing rapidly. In addition, given the data were captured after patients had used the device only for the second or third occasion (assuming they only using the training device once), it is possible that the sound of the device actuating may have influenced their manoeuvre.

## Conclusions

The NEXThaler multi-dose DPI has a BAM with a dose counter that activates solely following inhalation (so effectively an inhalation counter). The two studies demonstrated that patients with controlled and partly controlled/uncontrolled asthma, and those with a range of severities of COPD were able to trigger the BAM and consequently activate the inhalation counter, thus receiving feedback on inhalation.

## Supplementary Information

**Additional file 1.** Device usability questionnaire and results.

## Data Availability

Chiesi commits to sharing with qualified scientific and medical researchers, conducting legitimate research, the anonymised patient-level and study-level data, the clinical protocol and the full clinical study report of Chiesi Farmaceutici SpA-sponsored interventional clinical trials in patients for medicines and indications approved by the European Medicines Agency and/or the US Food and Drug Administration after 1st January 2015, following the approval of any received research proposal and the signature of a Data Sharing Agreement. Chiesi provides access to clinical trial information consistently with the principle of safeguarding commercially confidential information and patient privacy. Other information on Chiesi’s data sharing commitment, access and research request’s approval process are available in the Clinical Trial Transparency section of http://www.chiesi.com/en/research-and-development/, including the clinical trial data request portal.

## Abbreviations

BAM: Breath-actuated mechanism
COPD: Chronic obstructive pulmonary disease
DPI: Dry-powder inhaler
FEV_1_: Forced expiratory volume in 1 s
GOLD: Global Initiative for Chronic Obstructive Lung Disease
PEF: Peak expiratory flow
PIF: Peak inspiratory flow
