# Supplementary material for: Inspiratory flow profile and usability of the NEXThaler, a multidose dry powder inhaler, in asthma and COPD
Source: BMC Pulm Med. 2021 Feb 25;21:65. doi: 10.1186/s12890-021-01430-9 (PMC7905605; doi:10.1186/s12890-021-01430-9)
Supplement: Supplementary file 1 — Additional file 1. Device usability questionnaire and results. [file 12890_2021_1430_MOESM1_ESM.docx]

# Inspiratory flow profile and usability of the NEXThaler, a multidose dry powder inhaler, in asthma and COPD

Alfredo Chetta,^1^ Arzu Yorgancioglu,^2^ Mario Scuri,^3^ Sara Barile,^3^ Daniele Guastalla,^3^ P N Richard Dekhuijzen^4^

1. Respiratory Disease and Lung Function Unit, Department of Medicine and Surgery, University of Parma, Parma, Italy.

2. Department of Pulmonology, Medical Faculty, Celal Bayar University, Manisa, Turkey.

3. Chiesi Farmaceutici SpA, Parma, Italy.

4. Radboud University Medical Center, Nijmegen, The Netherlands.

## Supplement: Device usability questionnaire and results

## Questions included in the device usability questionnaire

- Was 5 min sufficient to train the patient?
- Was the device damaged during opening of the packaging?
- Was the inhalation counter appearance clear?
- Was a click heard when opening inhaler?
- Was a click heard soon after inhalation?
- Did the mouthpiece fit well?
- Did the patient block the air vents?
- Did the patient exhale into the device?
- Did the patient trap or cut their lips in the mouthpiece whilst inhaling?
- Were there any extreme environment effects on the inhaler?

Each question was answered yes or no, with the responses entered directly in the patient’s case report form.

## Results

Supplementary table 1. Asthma study: Device usability evaluation.

|  | **Asthma control** | | **Total  (N=40)** |
| --- | --- | --- | --- |
|  | **Controlled (N=20)** | **Partly controlled/ uncontrolled (N=20)** |  |
| Was 5 min sufficient to train the patient? n (%) | | | |
| Yes | 20 (100) | 20 (100) | 40 (100) |
| Was the device damaged during opening of the packaging? n (%) | | | |
| No | 20 (100) | 20 (100) | 40 (100) |
| Was the inhalation counter appearance clear? n (%) | | | |
| Yes | 19 (95.0) | 20 (100) | 39 (97.5) |
| No | 1 (5.0) | 0 | 1 (2.5) |
| Was a click heard when opening inhaler? n (%) | | | |
| Yes | 20 (100) | 20 (100) | 40 (100) |
| Was a click heard soon after inhalation? n (%) | | | |
| Yes | 20 (100) | 20 (100) | 40 (100) |
| Did the mouthpiece fit well? n (%) | | | |
| Yes | 20 (100) | 20 (100) | 40 (100) |
| Did the patient block the air vents? n (%) | | | |
| No | 20 (100) | 20 (100) | 40 (100) |
| Did the patient exhale into the device? n (%) | | | |
| No | 20 (100) | 20 (100) | 40 (100) |
| Did the patient trap or cut their lips in the mouthpiece whilst inhaling? n (%) | | | |
| No | 20 (100) | 20 (100) | 40 (100) |
| Were there any extreme environment effects on the inhaler? n (%) | | | |
| No | 20 (100) | 20 (100) | 40 (100) |

Supplementary table 2. COPD study: Device usability evaluation.

|  | **GOLD Grade** | | | | **Total**  **(N=69)** |
| --- | --- | --- | --- | --- | --- |
|  | **1**  **(N=19)** | **2**  **(N=20)** | **3**  **(N=20)** | **4**  **(N=10)** |  |
| Was 5 min sufficient to train the patient? n (%) | | | | | |
| Yes | 19 (100) | 20 (100) | 20 (100) | 10 (100) | 69 (100) |
| Was the device damaged during opening of the packaging? n (%) | | | | | |
| No | 19 (100) | 20 (100) | 20 (100) | 10 (100) | 69 (100) |
| Was the inhalation counter appearance clear? n (%) | | | | | |
| Yes | 19 (100) | 20 (100) | 20 (100) | 10 (100) | 69 (100) |
| Was a click heard when opening inhaler? n (%) | | | | | |
| Yes | 19 (100) | 20 (100) | 20 (100) | 10 (100) | 69 (100) |
| Was a click heard soon after inhalation? n (%) | | | | | |
| Yes | 19 (100) | 20 (100) | 20 (100) | 10 (100) | 69 (100) |
| Did the mouthpiece fit well? n (%) | | | | | |
| Yes | 19 (100) | 19 (95.0) | 20 (100) | 10 (100) | 68 (98.6) |
| No | 0 | 1 (5.0) | 0 | 0 | 1 (1.4) |
| Did the patient block the air vents? n (%) | | | | | |
| No | 19 (100) | 20 (100) | 20 (100) | 10 (100) | 69 (100) |
| Did the patient exhale into the device? n (%) | | | | | |
| No | 19 (100) | 20 (100) | 20 (100) | 10 (100) | 69 (100) |
| Did the patient trap or cut their lips in the mouthpiece whilst inhaling? n (%) | | | | | |
| No | 19 (100) | 20 (100) | 20 (100) | 10 (100) | 69 (100) |
| Were there any extreme environment effects on the inhaler? n (%) | | | | | |
| No | 19 (100) | 20 (100) | 20 (100) | 10 (100) | 69 (100) |

GOLD, Global Initiative for Chronic Obstructive Lung Disease.
